# Biological Therapy for Psoriasis in Cancer Patients: An 8-Year Retrospective Real-Life Study

**DOI:** 10.3390/jcm13071940

**Published:** 2024-03-27

**Authors:** Teresa Battista, Lucia Gallo, Fabrizio Martora, Davide Fattore, Luca Potestio, Sara Cacciapuoti, Massimiliano Scalvenzi, Matteo Megna

**Affiliations:** Section of Dermatology, Department of Clinical Medicine and Surgery, University of Naples Federico II, Via Pansini 5, 80131 Napoli, Italy; luciagallodr@gmail.com (L.G.); dott.davidefattore@gmail.com (D.F.); sara.cacciapuoti@libero.it (S.C.); mat24@libero.it (M.M.)

**Keywords:** psoriasis, biologic therapy, cancer

## Abstract

**Background**: It is now recognized that psoriasis plays a key role in the development of several comorbidities, such as cardiovascular disease, and metabolic syndrome. Some authors have hypothesized that patients with psoriasis may have an increased risk of developing certain types of cancer. The efficacy and safety of biologic drugs are well-documented in clinical trials and in real-life studies. However, there is limited evidence on the safety of the use of biologic treatments in cancer patients with psoriasis, and the use of this therapeutic class in patients with a pre-existing or concomitant malignancy is still debated. **Methods**: We have conducted a retrospective observational study of a group of oncology patients with moderate-to-severe psoriasis treated with biologic therapy at the Dermatology Clinic of the University of Naples Federico II, during the period from 2016 to 2024. We included 20 adult patients; in 15 of them the diagnosis of neoplasm preceded the start of treatment biologic, while four of these patients had been diagnosed with cancer during the course of therapy biologics. **Results**: The most represented neoplasms in our population were breast carcinoma, prostate carcinoma, thyroid carcinoma, and chronic lymphatic leukemia. Anti-IL17 drugs were the most frequently prescribed (47.7%), followed by anti-IL23p19 (36.8%), anti-IL-12/23 (10.5%) and anti-TNF alpha (5.26%). All patients showed improvement of psoriasis after starting the therapy. **Conclusions**: Our experience supports the effectiveness and safety of biological therapy for psoriasis in patients with a history of cancer or recent onset neoplasia.

## 1. Introduction

Psoriasis is a chronic T-cell-mediated skin disease that affects approximately 2–4% of the world’s population [1]. It is strongly associated with pathogenetically related immune-mediated inflammatory diseases, such as psoriatic arthritis (which affects up to 30% of psoriasis patients), inflammatory bowel disease and hidradenitis suppurativa [2]. It is now well-recognized that psoriasis also plays a key role in the development of several comorbidities, such as cardiovascular diseases and metabolic syndrome [2]. Common metabolic comorbidities of psoriasis include hypertension, diabetes, obesity, and hepatic steatosis, leading to an increased risk of cardiovascular disease [2]. Some authors hypothesized cancer as another possible comorbidity of psoriasis. In 2013, a systematic review and meta-analysis reported that patients with psoriasis may have an increased risk of certain solid tumors, lymphomas and keratinocyte tumors [3]. Since then, a number of larger studies have been carried out on the possible association between psoriasis and cancer [4,5,6,7,8]. Moreover, some studies have shown that psoriasis and cancer share the same exposome [9,10]. In a more recent systematic review and meta-analysis of 112 studies that included more than 2 million patients, the overall cancer risk was found to be mildly increased in patients with psoriasis, especially the risk of keratinocyte cancer and lymphomas [11]. The overall prevalence of cancer in patients with psoriasis was 4.78%, with an incidence rate of 11.75 per 1000 person-years and a risk ratio (RR) of 1.21. There was an increased risk of several cancers, including keratinocyte cancer (RR, 2.28), lymphomas (RR, 1.56), lung cancer (RR, 1.26) and bladder cancer (RR, 1.12). No increased risk of cancer for patients with psoriasis treated with biologic agents was found (RR, 0.97) [11]. Other studies suggest that psoriasis patients also have an elevated risk of developing cancers of the colorectum, kidney, larynx, liver, esophagus, oral cavity and pancreas [12,13]. The potential factors contributing to the connection between psoriasis and cancer are manifold, with lifestyle-related exposure to carcinogens playing a significant role. Factors such as tobacco smoking, alcohol abuse, and prolonged exposure to sunlight or ultraviolet light are more prevalent among psoriasis patients, potentially contributing to an elevated risk of lip, throat, lung, bladder, and non-melanoma skin cancers [4]. Additionally, central obesity and the presence of psoriasis itself, particularly if severe or accompanied by psoriatic arthritis, likely contribute to chronic systemic inflammation, which could predispose individuals to cancer development through the overexpression of multiple cytokines [4]. The risk of developing keratinocyte cancer is notably associated with sunlight exposure. Studies examining the risk of keratinocyte cancer in patients undergoing psoralen-UVA (PUVA) therapy indicate a markedly increased risk, particularly concerning squamous cell carcinomas [14]. Furthermore, a separate study assessed the risk of melanoma and keratinocyte cancer among psoriasis patients compared to the general population in Denmark [6]. When adjusting for confounders, including PUVA, the study found a small but significant increase in the risk of keratinocyte cancer, indicating that factors other than PUVA treatment may be important. Patients with psoriasis visit the dermatologist more often than the general population and, hence, more keratinocyte cancers may be found [15]. Concerning lymphomas, studies have shown that patients with psoriasis have an increased risk of developing Hodgkin’s and non-Hodgkin’s lymphoma [16]. This increase could be partly explained by an increased risk of cutaneous T-cell lymphoma (CTCL) in patients with psoriasis due to a sustained immune activation, which could result in the emergence of a dominant clone. Additionally, the influence of immunosuppressive treatments may play a role in this association [16].

These data are influenced by issues of differential diagnosis between the psoriasis and CTCL since an early stage of mycosis fungoides can be frequently mistaken for psoriasis; indeed, both diseases present with erythematous-desquamative patches and/or plaques [16]. Therefore, a potential overestimation of the association between psoriasis and mycosis fungoides may exist. When psoriasis becomes refractory to topical treatments, systemic therapies become necessary. Immunosuppressants like methotrexate and cyclosporine are crucial in managing these cases. However, cyclosporine is contraindicated in patients with a history of cancer. Retinoids are considered safe for cancer patients but should be avoided in individuals with metabolic syndrome [17]. Treatment with apremilast is also advised for cancer patients, although safety data are extrapolated from physiological considerations due to the lack of long-term studies in populations with previous malignancies [17].

In severe or refractory psoriasis, guidelines recommend using biological therapies directed against TNF-alpha (adalimumab, etanercept, certolizumab, infliximab) or the IL-23/17 axis (ustekinumab, guselkumab, risankizumab, tildrakizumab, brodalumab, ixekizumab, secukinumab, bimekizumab) [18,19]. The efficacy and safety of biological agents are well-documented in clinical trials and also in real life [20,21,22]. However, the evidence of safety for the use of biologic treatments in psoriasis patients with a history or ongoing malignancy is scarce, and the use of this therapeutic class in patients with a prior or concomitant malignancy is still debated. National and international guidelines recommend the avoidance of biologic drug use in patients with a history of malignancy within the past 5 years [23,24]. Recently released European guidelines propose taking into account the risk of neoplastic disease progression and recurrence, as well as the impact of psoriasis burden, when making treatment decisions. They also advocate for individualized discussions with an oncologist prior to initiating biological therapy [24].

The use of biologic drugs has demonstrated their ability to induce the regression of symptoms in psoriasis patients. However, their role in patients with concurrent cancer diagnoses remains a topic of ongoing debate. This uncertainty results in many individuals with severe psoriasis and/or psoriatic arthritis being excluded from the potential therapeutic benefits of biologic drugs, despite the substantial impact these conditions have on their quality of life. Notably, patients with a history of cancer who also suffer from psoriasis represent a population with unmet medical needs [25]. The influence of IL 12, 23, and 17, as well as their inhibition, on tumor physiology is complex and not fully understood. Similarly, the role of TNF alpha inhibition in the progression of neoplastic diseases is a subject of questioning [26,27,28]. Furthermore, there are limited studies available that can accurately assess the risk of cancer progression or recurrence in psoriasis patients receiving biologic drug treatments. In the absence of large, well-structured studies specifically focusing on this patient population, real-life experiences and expert opinions become particularly valuable resources for clinicians seeking to make informed therapeutic decisions. This study aims to provide insight into the experiences of a group of patients dealing with moderate-to-severe psoriasis and a history of previous or concurrent cancer who have been treated with biologic drugs. In addition to our own experience, we conducted a systematic review to highlight the current existing literature, shedding light on the safety and efficacy of biologic drugs in this unique patient population.

## 2. Methods

### 2.1. Case Series

We conducted an extensive review of our records and performed a retrospective observational study to identify all instances of psoriasis patients treated with biologic drugs who had either a preexisting or newly diagnosed cancer at the Dermatology Clinic of the University of Naples Federico II, spanning from 2016 to 2024. This study obtained approval from the Ethics Review Committee of the University of Naples Federico II, and patients provided consent for the anonymous publication of their data. Our center follows the Italian Guidelines for psoriasis treatment [23].

### 2.2. Literature Review

We conducted a thorough search of MEDLINE/PubMed from its inception until November 2023. The search terms ‘psoriasis’ were combined using the Boolean operator ‘AND’ with ‘adalimumab’, ‘etanercept’, ‘infliximab’, ‘certolizumab’, ‘secukinumab’, ‘ixekizumab’, ‘brodalumab’, ‘bimekizumab’, ‘guselkumab’, ‘tildrakizumab’, and ‘risankizumab’. These terms were also combined with ‘cancer’, ‘malignancy’, ‘neoplasm’, and ‘carcinoma’. Additionally, a recent systematic review was conducted using PubMed and SCOPUS, which identified 272 previously published cases, to which the authors’ institution contributed an additional 19 cases [26,27,29,30,31,32,33,34,35,36,37,38,39,40,41,42,43]. Eligible studies were those reporting the use of biologic drugs in patients with psoriasis or psoriatic arthritis who had a history of previous or concurrent malignancy. These studies needed to provide specific information such as the type of tumor, the biologic drug used, the time interval between tumor diagnosis and the initiation of biologic treatment, follow-up periods, and any instances of cancer recurrence, progression, or the emergence of new malignancies. These details constituted the primary outcomes of the research. The screening process involved assessing the title and abstract of the articles. Articles deemed relevant based on these initial assessments were then thoroughly reviewed in their entirety. Only articles meeting the predefined inclusion and exclusion criteria were selected for further analysis. For each included study, the following information was extracted: the gender and age of the patient at the time of cancer diagnosis, the duration between cancer diagnosis and the initiation of biologic treatment, the various types of biologics administered following the cancer diagnosis, the duration of follow-up since the commencement of the first biologic treatment, and any reported cases of cancer progression, recurrence of prior malignancies, or the emergence of additional cancer cases.

## 3. Results

We retrieved the electronic records of 20 patients (12 women and eight men) whose ages ranged from 36 to 75 years, as shown in Table 1. Eleven patients (60%) had previously undergone non-biologic systemic treatments (such as cyclosporine, methotrexate and acitretin) and/or phototherapy prior to commencing biologic therapy.

The patients in our study exhibited diverse cancer types and locations, as detailed in Table 1. 

Fifteen patients (75%) of our case series had received a cancer diagnosis prior to the commencement of biologic treatment, falling into the ‘precedent cancer’ (PC) group. In our patient group, the mean duration from cancer diagnosis to the initiation of biological therapy is 3.8 ± 3 years. For the remaining four patients (20%), cancer was diagnosed while undergoing biologic treatment for psoriasis, placing them in the ‘intercurrent cancer’ (IC group).

In the PC group, all individuals had achieved remission at the initiation of biologic therapy. The time elapsed between tumor diagnosis and the commencement of biological therapy ranged from 12 to 84 months.

Regarding the PC group, eight patients (53.3%) received anti-IL-17 biologics, six patients (40%) were treated with anti-IL-23 agents, and one patient (6.7%) received treatment with anti-IL-12/23.

In the IC group (*n* = 4), the biologics prescribed prior to cancer diagnosis included anti-TNFα in two patients (50%), anti-IL-12/23 in one patient (25%), and anti-IL-23 in the remaining patient.

In all those patients the biologic treatment was discontinued due to safety concerns after the cancer diagnosis and the treatment was resumed upon the favorable opinion of the oncologist. Among these patients, two patients were treated with the same biological drugs previously suspended (certolizumab and ustekinumab), while the other two patients switched, one from anti-IL-23 to anti-IL-17 and the other from anti-TNF to anti-IL-17. Biological therapy was resumed an average of 18 months after discontinuation following tumor diagnosis, ranging from 12 to 24 months.

The median follow-up duration after biologic start to resume was 30.2 ± 21.8 months, with a range of 1 to 84.

Among the 20 patients included in our study, the median PASI value at biologic initiation was 31.3. In terms of biologic efficacy, all patients in this group exhibited a significant amelioration in psoriasis following biologic initiation.

Out of the 20 patients included, only one patient, the one with oesophageal cancer being treated with ustekinumab, experienced cancer progression and will restart chemotherapy. No additional severe adverse events, and none associated with biologic treatment, were reported throughout the follow-up period.

## 4. Discussion

The potential link between psoriasis and cancer is influenced by a myriad of factors, with lifestyle-related exposure to carcinogens playing a prominent role. Individuals with psoriasis often exhibit higher rates of tobacco smoking, alcohol consumption, and prolonged sun/ultraviolet light exposure, contributing to their elevated susceptibility to developing lip, oral, throat, lung, bladder, and non-melanoma skin cancers. Furthermore, obesity, prevalent among psoriasis patients, has been associated with various cancers, including colorectal cancer [12,13]. Central obesity, in conjunction with psoriasis itself, particularly when severe or accompanied by psoriatic arthritis, may fuel systemic chronic inflammation, potentially predisposing individuals to cancer development through the overexpression of multiple cytokines [12].

Certain treatments for psoriasis have also been implicated as potential carcinogens. Although tar-containing topical treatments are no longer utilized, current corticosteroid and calcipotriene formulations are generally regarded as non-carcinogenic. However, photo(chemo)therapy involving 8-methoxypsoralen plus ultraviolet A (PUVA) and narrow-band ultraviolet B (nb-UVB) has been linked to cutaneous malignancies, exhibiting a dose-dependent pattern [44]. Prolonged iatrogenic immunosuppression is a well-established risk factor for cancer development and progression [44]. Immunosuppressive agents such as methotrexate and cyclosporine are commonly employed in psoriasis treatment. Methotrexate has been associated with a slight elevation in melanoma risk, though the absolute increase is considered minimal [45]. Cyclosporine is linked with various side effects, including nephrotoxicity, hypertension, and complications of immunosuppression such as heightened susceptibility to infection and malignancy. The augmented risk of cutaneous malignancy in organ transplant recipients receiving higher CsA doses has been extensively documented [46].

The risk associated with the use of cyclosporine in psoriasis treatment appears to be limited, given its administration at relatively low concentrations and for short durations. However, an elevated risk of both melanoma and non-melanoma skin cancers (NMSC) has been observed in patients undergoing PUVA treatment. Paul et al. conducted a prospective study involving 1252 psoriasis patients treated with cyclosporine for up to 5 years, reporting an increased overall risk of malignancy compared to the general population. This increased risk was primarily attributed to a six-fold higher incidence of skin malignancies, particularly squamous cell carcinoma, which was influenced by prolonged treatment durations (>2 years) and prior therapies (PUVA and methotrexate) [47], thus confirming the heightened risk of squamous cell carcinoma in cyclosporine-treated patients, particularly following exposure to PUVA [13,48,49].

Furthermore, several cases in the literature highlight a higher incidence of lymphoproliferative disorders in patients undergoing prolonged and higher-dose cyclosporine therapy [29,50,51].

Acitretin and fumaric acid esters have not demonstrated an increased risk of cancer development [44]. In fact, acitretin has been suggested for preventing skin cancer development in high-risk patients [13]. The use of apremilast, which selectively inhibits phosphodiesterase 4, is also considered safe for cancer patients [52]. In fact, apremilast is also integrated into the therapeutic regimen for treating immune checkpoint inhibitor (ICI)-mediated psoriasis [53].

Biologic targeted therapies for immune-mediated inflammatory diseases have been closely monitored since their approval, primarily due to initial safety concerns [11]. Some studies have proposed a potential association between anti-TNFα biologic treatment and an increased risk of lymphoma [54,55]. Additionally, a comprehensive review of the literature prior to 2009 suggested that anti-TNFα treatments in psoriasis might carry an elevated risk of cancer, particularly non-melanoma skin cancers and hematologic malignancies [55]. However, a study involving long-standing, incident, and anti-TNFα-treated cohorts of rheumatoid arthritis patients, linked with the Swedish Cancer Registry, concluded that the risk of lymphoma in anti-TNFα-treated patients was not higher compared to other cohorts [56,57]. The PSOLAR registry indicated that long-term exposure to anti-TNFα treatments might increase cancer risk (OR 1.54), but this increase was not statistically significant when analyzed by individual anti-TNFα agents [58]. Furthermore, no significant increase in cancer risk was observed after seven years of adalimumab treatment among patients included in the ESPRIT registry [30,59,60]. Lastly, an extensive systematic review and meta-analysis published in 2020 found no significant differences in cancer incidence between patients with psoriasis treated with biologics and those treated with non-biologic therapies (RR, 0.97) [11].

The heightened risk of cancer in patients with psoriatic disease may stem from a complex interplay of factors, including lifestyle-related exposure to carcinogens and the systemic inflammation triggered by psoriasis. This inflammatory milieu leads to the upregulation of proinflammatory cytokines, such as TNF-α, IL-1β, IL-12, IL-17, IL-22, and IL-23 [13]. Elevated levels of these inflammatory cytokines, particularly IL-6, TNF-α, and IL-1β, can induce epigenetic alterations in cells, resembling the activation of oncogenes and the suppression of tumor suppressor genes [13]. Additionally, chronic inflammation has been associated with the activation of signaling pathways like signal transducer and activator of transcription 3 (STAT3) and nuclear factor-kappaB (NF-kB). Pro-tumorigenic cytokines like IL-17, IL-21, IL-22, IL-23, and IL-6 activate STAT3 transcription factors, while NF-kB is activated by cytokines like IL-17, IL-23, TNFα, IL-1, and IL-18. STAT3 and NF-kB promote the activation of anti-apoptotic genes in tumor cells [13].

Despite its name, TNFα was initially anticipated to revolutionize cancer treatment upon its discovery. However, accumulating evidence over the years suggests both anti-tumoral and pro-tumoral effects of TNFα, contingent on factors such as the cellular source (tumoral or immune), the target cell, the type of TNFα receptor engaged, and the microenvironment in which it is released [30,31,32,33]. As previously mentioned, anti-TNFα agents have been linked to lymphoma, yet they are safely administered to patients experiencing moderate-to-severe chemotherapy-induced side effects [52,59]. Initially recognized for its ability to induce rapid hemorrhagic necrosis in experimental cancers, TNFα has demonstrated a paradoxical role in promoting tumors as endeavors to exploit its anti-tumor activity progressed [28]. Recent studies on murine models have underscored that TNFα itself is produced by tumor cells, inducing increased production of growth factors, stimulation of angiogenesis, and enhanced capillary permeability [28].

TNFα is also known to impede the infiltration of tumors by CD8+ lymphocytes, trigger activation-induced cell death of CD8+ T cells, and inhibit the responses of cytotoxic CD8+ T cells, thereby fostering immune evasion and relapse in melanoma. TNFα additionally enhances the expression of anti-PD-L1 [34,35,36,37]. Moreover, anti-PD-1 treatments can induce the production of TNFα, impairing therapeutic responses in melanoma, lung, and breast cancer in murine models [33]. The combination of anti-PD-1 and anti-TNFα has demonstrated superior outcomes and therapeutic relevance compared to anti-PD-1 alone, providing robust evidence supporting this approach for the treatment of certain malignancies in the near future [33]. Recent meta-analyses and observational studies have found no significantly increased risk of systemic malignancies, including lymphoma, with anti-TNF-α in psoriasis. Conversely, several recent studies have confirmed an increased risk of squamous cell carcinoma [36,37,38].

As for ustekinumab, which targets the shared p40 subunit of both IL-12 and IL-23, its product information cautions against its use in patients with a history of cancer. IL-12 is believed to promote tumor suppression by facilitating the infiltration of cytotoxic T cells, while IL-23 promotes Th17 immunity, potentially contributing to tumor development. The balance between IL-12 and IL-23 plays a crucial role in carcinogenesis [39]. Additionally, IL-23 is thought to play a significant role in gut tumorigenesis [39].

Regarding IL-17, numerous investigations have explored its potential role in tumorigenesis [27]. Chronic production of IL-17 has been associated with the initiation, growth, and metastasis of tumors in various malignancies [27]. Recently, it has been demonstrated that IL-17 can directly influence tissue stem cells, promoting tissue repair and tumorigenesis [27,40]. Moreover, elevated levels of IL-17 have been linked to poor prognosis in various solid tumors [27]. In preclinical cancer models, inhibiting IL-17 has led to the suppression of metastasis and improved responses to chemotherapy and radiotherapy [27]. Consequently, IL-17 inhibitors have been proposed as adjuvant treatments to prevent resistance to chemotherapy and radiotherapy [26,40].

European guidelines for the systemic treatment of moderate-to-severe psoriasis vulgaris suggest considering anti-TNFα agents, ustekinumab, IL-17, and IL-23 inhibitors for patients with cancer, with individualized assessments considering patient preferences and remission durations. Furthermore, consensus guidelines from the British Society of Gastroenterology for the management of inflammatory bowel disease in adults do not contraindicate biologic agents in patients with a history of cancer. However, they recommend a 2-year delay following cancer remission, which should be extended to 5 years for cancers with a high risk of early dissemination and late metastasis, such as breast cancer, malignant melanoma, and renal cell carcinoma [41].

In conclusion, the inhibition of IL-23 and IL-17, alongside their therapeutic effects in psoriasis, may confer benefits to patients with previous or concomitant cancer. Conversely, conflicting data exist in the literature regarding the safety of anti-TNFα and anti-IL-12/23 in psoriasis patients with cancer [26,27,36,39,40,41,42,43,59,61]. We now present data on 20 patients with moderate to severe psoriasis who had cancer before or concurrently with biological treatment. The treatment was generally effective and consistently safe. In our patient group, the most frequently used biologic was secukinumab [six out of 20 patients (30%)], followed by guselkumab [four out of 20 patients (20%)]. The average follow-up duration is 30.2 ± 21.8 months, during which no tumor recurrences, progression, or new cancer diagnoses occurred in our patient group. Only one patient experienced a recurrence of esophageal tumor while undergoing ustekinumab therapy, leading to subsequent resumption of chemotherapy. Our experience aligns with a systematic review encompassing 272 psoriasis patients treated with biologics who had a history of or concurrent cancer [26,27,33,34,35,36,37,38,39,40,41,42,43,54,61,62,63,64,65,66,67] (see Table 2).

In randomized double-blinded placebo-controlled trials, namely VOYAGE 1 and 2 trials, the efficacy and safety of the IL-23 agent guselkumab were assessed against placebo and adalimumab. Patients with a cancer history without recurrence for more than 5 years before screening were enrolled [62]. Out of 1826 patients, 20 patients with a history of malignancy were identified. Among the 20 patients exposed to guselkumab, only two malignancies were reported. One case involved a new primary malignancy—metastatic breast cancer diagnosed in a patient with a history of previous prostate cancer, leading to study withdrawal. The second case involved the recurrence of their primary malignancy (lung cancer), which was fatal. Non-melanoma skin cancers were reported in two patients. Blauvelt et al. emphasized the need for further investigation with larger sample sizes and longer follow-up to gain a better understanding of the future malignancy risk associated with biologics for dermatology patients with a history of cancer [62].

To date, the largest prospective study by van Lümig et al. included 173 psoriatic patients, some with a previous history of cancer. However, only a small number of such patients were included, and complete information was available for three of them. This study did not observe recurrences of solid tumors, and only one female patient with a previous history of non-melanoma skin cancers developed new basal cell carcinomas [33]. The largest case series, involving 14 psoriatic patients, was reported by Odorici et al. [63]. In this study, biologic drugs were used to treat psoriasis in patients with advanced-stage tumors, including one lung adenocarcinoma, one renal carcinoma, and one rectal adenocarcinoma. No recurrence or progression was detected, but three different patients experienced the onset of multiple non-melanoma skin cancers, one small-cell lung carcinoma, and myeloma. Bellinato et al. reported a substantial case series of 12 psoriatic patients with a previous and concurrent history of cancers treated with anti-IL17. The reported malignancies included three cases of melanoma, two cases of prostate carcinoma, two cases of breast cancer, and one case each of multiple myeloma, rectal carcinoma, and bladder carcinoma. The majority of the tumors were in the early stages. One patient, previously treated for breast cancer with radiotherapy, developed two in situ squamous cell carcinomas of the skin. Another patient, with a history of melanoma, developed a small-cell lung carcinoma during treatment with secukinumab [64].

Mastorino et al. presented a series of 37 real-world patients with psoriasis who had a history of cancer and were subsequently treated with biologics [65]. Reported malignancies encompassed a diverse range of cancers, including breast cancer, melanoma, basal cell carcinoma, prostate cancer, meningioma, renal cancer, colon cancer, bladder cancer, cutaneous squamous cell carcinoma, seminoma, larynx cancer, endometrial cancer, ovarian cancer, thyroid cancer, ameloblastoma, leiomyosarcoma, and acute lymphocyte leukemia. Most of the cases were early-stage neoplasms. They found that biologic treatments were safe in this special population [65]. Among the patients who underwent regular follow-up, only one patient with endometrial cancer experienced progression during treatment. The progression was deemed a natural evolution of the patient’s tumor, and consequently, the biologic treatment was not interrupted [65].

Another real-life study reported the experience of 16 patients with a history of cancer treated with biologic drugs [66]. Four of these patients had a previous medical history of breast cancer, three patients had a history of Non-Hodgkin Lymphoma, two had kidney cancer, one had thyroid cancer, one had both pheochromocytoma and thyroid cancer, one had lung cancer, one had brain cancer, one had intestinal cancer, and one had uterine cancer. Eight patients received treatment with anti-IL-17 agents (five with ixekizumab and three with secukinumab), four patients were treated with ustekinumab (anti-IL12/23), two with etanercept (anti-TNFalpha), and two with anti-IL23 drugs (one with risankizumab and one with guselkumab). Among these patients, all had a history of cancer within the previous 10 years, with five out of the 16 patients having received a cancer diagnosis within the past 5 years. No disease recurrence or new tumors were observed during the entire follow-up period, which extended to 96 weeks for nine patients and 144 weeks for the remaining seven patients [66].

Rusiñol et al. recently conducted a study involving 31 patients with moderate to severe psoriasis who had a history of cancer prior to or concurrent with biologic treatment [67]. The treatment was generally effective and consistently safe. Among these patients, there were various types of cancer, including genitourinary cancers (four with prostate cancer, two with renal cell carcinoma, and two with bladder cancer), breast cancer (six patients), and hematologic malignancies (two with follicular lymphoma, two with chronic lymphocytic leukemia, one with diffuse large B cell lymphoma, and one with monoclonal gammopathy of undetermined significance). Additionally, there were cases of colorectal carcinoma, thyroid carcinoma, melanoma, gastric carcinoma, head and neck cancer, leiomyosarcoma, and chondrosarcoma. Out of the 31 patients, 16 (52%) were diagnosed with cancer before the initiation of biologic treatment. In the remaining 15 patients, cancer was diagnosed during the course of biologic treatment. Among the 16 patients of the first group, nine (56%) had achieved remission without oncologic treatment when biologic therapy for psoriasis began. The duration of remission ranged from one to more than 5 years, with seven of the nine patients having been in remission for more than 5 years. In the group with a history of previous cancer, five patients received anti-TNFα treatment, seven received anti-IL-23 biologics, three were treated with anti-IL-17 agents, and one patient received ustekinumab. Among the seven patients who were under oncologic treatment when biologic therapy was initiated, five were treated with anti-IL-23 agents, one with an IL-17 inhibitor, and one received anti-TNFα treatment. In the entire cohort of 31 patients, only four experienced cancer progression despite ongoing oncologic treatment, and tragically, two of them did not survive.

Finnegan et al. conducted a case series on 11 patients diagnosed with malignancy while on biologic therapies [68]. Biologics were prescribed for the treatment of: psoriasis (*n* = 9), hidradenitis suppurativa (HS) (*n* = 1), and dual pathology of cutaneous Crohn’s disease and HS. In nine of these patients biologics therapy was halted and in all of them psoriasis flared. As recommended by European guidelines, topical therapy, phototherapy, and acitretin were administered. However, two patients, one diagnosed with breast cancer and one with non-Hodgkin’s lymphoma, resumed biological therapy two years after their cancer diagnosis, due to the stubborn psoriasis. The authors highlighted the significant clinical challenge of managing these patients that necessitated multiple consecutive agents to control their disease.

Pellegrini et al. [69] undertook a multicenter real-life study that specifically examined a particular biologic, contributing further evidence to the safety profile of secukinumab in psoriatic patients with a history of cancer. The study included forty-two patients, with malignancy diagnosed within the past 5 years in 21 (56.8%) and within the past 10 years in 37 (88.1%). Eight (19.0%) patients had a previous diagnosis of melanoma, seven (16.7%) patients each had breast and colorectal cancer, five (11.9%) bladder cancer, five (11.9%) lung cancer, four (9.5%) prostate cancer, three (7.1%) kidney cancer, and one patient (2.4%) each cancer of the uterus, testicle, and lymphoma. Over an average treatment period of 56 ± 31.7 weeks, there were no instances of tumor recurrence or progression. However, three patients developed new malignancies unrelated to their previous cancer.

Recently, Magnano et al. [70] conducted a multicenter retrospective study on 73 psoriatic patients, categorized into three groups: Group 1 with a concurrent cancer diagnosis while undergoing biologic drug therapy, Group 2 with a recent cancer diagnosis, and Group 3 with a cancer history exceeding 5 years. The study compared patients receiving biologics before and after 5 years from the diagnosis of malignancy. During a comparable follow-up duration, there were no statistically significant variances found in terms of disease relapses (*p*-value = 0.66). Furthermore, in the literature, there are case reports of patients with a history or recent diagnosis of neoplasia who were effectively and safely treated with biologic drugs [71,72,73,74,75,76,77,78,79,80,81].

Our population is predominantly composed of females (12 patients out of 20, 60%). The most frequently encountered tumor in our population was breast cancer (six patients, 30%). In our experience, the most prescribed drugs were anti-IL-17 and anti-IL-23, with secukinumab ranking first (five patients, 25%). This is in line with literature data emphasizing the pro-tumorigenic role of IL-17 and IL-23 cytokines and the potential of their inhibitors in cancer therapy [82]. There has been only one recurrence and no new tumor occurrences during our follow-up. The average follow-up is 31.8 months, while the average time between cancer diagnosis and the start or resumption of biological therapy was 43.8 months.

## 5. Conclusions

Biologics have been used in the treatment of psoriasis for nearly 20 years now; the introduction of each newer class has been associated with increased efficacy and safety, but some concerns remain that limit their use in oncologic patients. These patients have been excluded from randomized clinical trials, but their numbers can be considerable, especially in hospital-based dermatology practices. Our real-life experience of 20 patients, although not a substitute for guidelines, supports the efficacy and safety of biologic treatments of psoriasis in patients with prior or intercurrent cancer and adds to the 272 additional cases found in our literature review. In conclusion, the safety of biologic drugs in psoriasis patients with a previous or concurrent cancer diagnosis remains a subject of interest and concern. While the evidence is limited, our real-world experience and literature review suggest that these treatments can be effective and safe for this special patient population. The conventional practice of waiting five years before initiating biologic therapy in patients with a history of cancer may be reconsidered, particularly in cases of severe skin disease. However, further research is needed to better understand the safety and efficacy of biologic drugs in patients with concurrent cancer, and to provide more definitive guidance for clinicians. The collaboration with oncology specialists and careful follow-up are crucial, especially for those undergoing active cancer treatment. As the population ages and the prevalence of oncological conditions rises, addressing the unique needs of psoriasis patients with cancer histories will become an increasingly important aspect of future medical practice. This study presents some limits, such as the small sample size, the retrospective analysis, the heterogeneity of tumors and treatments reported and the short duration of follow-up. In summary, we wish to highlight our favorable experience, which serves to validate the safety and efficacy of biologic medications in this specific cohort of psoriatic individuals. Additional multicenter investigations and clinical trials could provide valuable corroboration for our findings. 

## Figures and Tables

**Table 1:** Characteristics of cancer patients treated with biologics for psoriasis in our clinic. Abbreviations: FUP, follow up; NA, not available.

Patient Number	Age	Sex	Cancer Onset	Cancer	Year of Cancer Diagnosis	Stage	Treatment	FUP	PASI	Previous Non- Biologic Systemic Treatment	Previous Biologic Treatment	Therapy Discontinuation after Tumor Diagnosis (Months)	Date of First Biologic Therapy after Cancer	Ongoing Biologic Therapy and Start Date	Months from Cancer Diagnosis and Biologic Therapy	FUP under Biologic after Cancer Diagnosis (Months)
1	62	F	45	Endometrial cancer	2006	I	Surgery + RT	Regular	25	Methotrexate	None	None	2018	Ixekizumab (2018)	144	60
2	42	F	40	Breast cancer	2021	I	Surgery + RT + OT	Regular	11.3	None	None	None	2022	Secukinumab (2022)	6	20
3	65	M	59	Esophageal cancer	2016	IIA	Surgery + CT	Cancer progression	14.5	Methotrexate, Cyclosporine	Infliximab, Adalimumab, Ustekinumab	18	2018	Ustekinumab (2018)	18	84
4	48	F	45	Breast cancer	2020	I	Surgery	Regular	30.2	Methotrexate, Cyclosporine, Phototherapy	Adalimumab, Etanercept, Ustekinumab, Certolizumab	18	2022	Certolizumab (2022)	18	20
5	30	F	28	Thyroid cancer	2021	I	Surgery	Regular	18.3	Apremilast	None	None	2022	Tildrakizumab (2022)	12	24
6	67	F	66	Chronic lymphatic leukemia	2022	II	FUP	Regular	15.6	None	None	None	2022	Risankizumab (2022)	6	12
7	55	F	48	Chronic lymphatic leukemia	2018	I	FUP	Regular	20.4	Methotrexate	None	None	2018	Ustekinumab (2018)	3	60
8	65	M	63	Pulmonary cancer	2021	IV	Surgery + RT + CT	Regular	12.5	None	Guselkumab	12	2022	Secukinumab (2022)	12	18
9	52	F	45	Breast cancer	2016	IIIA	Surgery + RT + CT	Regular	8.2 (palmo-plantar)	Cyclosporine	None	None	2021	Ixekizumab (2021)	60	30
10	68	M	63	Prostate cancer	2018	II	Surgery + RT	Regular	24.6	None	None	None	2023	Risankizumab (2023)	60	6
11	70	F	60	Breast cancer	2013	NA	Surgery + RT + CT	Regular	9.3 (palmo-plantar and genitals)	Methotrexate, Cyclosporine	None	None	2019	Secukinumab (2019)	72	48
12	73	F	65	Breast cancer	2015	IIA	Surgery + RT + OT	Regular	19.5	Methotrexate	None	None	2021	Guselkumab (2021)	72	24
13	53	M	50	Thyroid cancer	2020	NA	Surgery + RT	Regular	35.7	Acitretin	None	None	2022	Secukinumab (2022)	24	12
14	66	M	60	Prostate cancer	2017	II	Surgery + RT	Regular	23.8	None	Adalimumab	24	2022	Ixekizumab (2022)	60	24
15	75	F	64	Breast cancer	2014	IV	Surgery + RT + CT	Regular	40.1	Methotrexate	None	None	2019	Guselkumab (2019)	60	48
16	36	F	29	Thyroid cancer	2016	II	Surgery + RT	Regular	17.5	None	None	None	2022	Secukinumab (2022)	72	24
17	63	M	58	Colo-rectal cancer	2018	NA	Surgery	Regular	22.3	Acitretin, methotrexate	None	None	2023	Guselkumab (2023)	6	12
18	61	M	52	Prostate cancer	2014	I	Surgery	Regular	18.7	Methotrexate, phototherapy	None	None	2019	Guselkumab (2019)	84	48
19	52	F	47	Breast cancer	2017	IIIA	Surgery + RT + CT	Regular	19	None	None	None	2023	Secukinumab (2023)	72	1
20	70	M	69	Pulmonary cancer	2023	III	CT + RT	Regular	50	None	Guselkumab	12	2024	Guselkumab (2024)	12	1

**Table 2:** Studies in the literature on the safety of biologic drugs in patients with psoriasis and history of cancer. Abbreviation: NA, not available.

Study	Biologic Drug (s)	Number of Patients Diagnosed with Cancer	Cancer Diagnosed	Average Time (Months) from Diagnosis to Biologics
Blauvelt et al. [62]	Guselkumab	2	Breast cancer Lung cancer	NA
Van Lümig et al. [33]	Alefacept Etanercept Adalimumab	4	Breast cancer	120
Odorici et al. [63]	Infliximab Etanercept Adalimumab Ustekinumab Secukinumab Efalizumab	14	Melanoma (2) Prostate carcinoma (4) Bladder adenocarcinoma (2) Lung adenocarcinoma (3) Rectal adenocarcinoma (2) Renal carcinoma (1) Breast adenocarcinoma (1) Non-melanoma skin cancers (1), myeloma (1)	40.1
Bellinato et al. [64]	Ixekizumab Secukinumab	12	Melanoma (1) Lung cancer (2) Prostate acinar adenocarcinoma (3) Breast ductal carcinoma (1) Bladder cancer (1) Colon adenocarcinoma (2) Uterus adenocarcinoma (1) Thyroid medullary carcinoma (1)	16.8
Mastorino et al. [65]	Guselkumab Brodalumab Secukinumab Ixekizumab Adalimumab Risankizumab	37	Leiomyosarcoma (1) Renal cancer (3) Ovarian cancer (1) Thyroid cancer (1) Larynx cancer (1) Non-melanoma skin cancer	112.2
Valenti et al. [66]	Ixekizumab Secukinumab Ustekinumab Etanercept Risankizumab Guselkumab	16	Breast cancer (4) Non-Hodgkin Lymphoma (3) Renal cancer (2) Thyroid cancer (1) Both pheochromocytoma and thyroid cancer (1) Lung cancer (1) Brain cancer (1) Intestinal cancer (1) Uterine cancer (1)	52
Rusiñol et al. [67]	Etanercept Adalimumab Ixekizumab Secukinumab Brodalumab Ustekinumab Risankizumab Guselkumab Tildrakizumab	31	Prostate cancer (4) Renal carcinoma (2) Bladder cancer (2) Breast cancer (6) Follicular lymphoma (2) Chronic lymphocytic leukemia (2) Diffuse large B cell lymphoma (1) Monoclonal gammopathy of undetermined significance (1) Colorectal carcinoma (1) Thyroid carcinoma (1) Melanoma (1) Gastric carcinoma (1) Head and neck cancer (1) Leiomyosarcoma (1) Chondrosarcoma (1)	NA
Finnegan et al. [68]	Adalimumab Etanercept Ustekinumab	11	Breast cancer (1) Bladder cancer (1) Ovarian cancer (2) Lymphoma (3) Gastric cancer (1) Esophageal cancer (1) Melanoma (1) Testicular cancer	24
Pellegrini et al. [69]	Secukinumab	43	Melanoma (8) Breast cancer (7) Bladder cancer (5) Colon cancer (7) Renal cancer (3) Lung cancer (5) Uterine cancer (1) Testicular cancer (1) Hodgkin lymphoma (1)	42
Magnano et al. [70]		73	Breast cancer Lymphomas Prostate cancer Kidney cancer Lung cancer Colorectal cancer Bladder cancer Uterus cancer Thyroid cancer Larynx cancer Melanoma Testicular cancers Non-melanoma skin cancer Hepatocarcinoma	28.06 (Group 2: Patients with recent diagnosis of malignancy) 109.2 (Group 3: Patients with malignancies who started a biologic after 5 years)
Balato et al. [71]	Etanercept Infliximab	1	Chronic lymphocytic leukemia	0
Patel et al. [72]	Etanercept Ustekinumab	1	Colon cancer	13
Lasagni et al. [73]	Secukinumab Ustekinumab	1	Breast cancer	22
Gkalpakiotos et al. [74]	Etanercept Ustekinumab	1	Melanoma	84
Ghazanfar et al. [75]	Secukinumab Ustekinumab	1	Melanoma	7
Kamiya et al. [76]	Guselkumab	1	Lung cancer	2
Wang et al. [77]	Ustekinumab	1	Kaposi sarcoma	18
Gambardella et al. [78]	Secukinumab	1	Bladder cancer	12
Jin et al. [79]	Ixekizumab	1	Multiple myeloma	0
Peter et al. [80]	Secukinumab	1	Melanoma	NA
Porcar Saura et al. [81]	Etanercept Adalimumab Ixekizumab	1	Laryngeal squamous cell carcinoma	5

## Data Availability

The data presented in this study are available on request from the corresponding author.
